# Development of MEDI4736, an anti-programmed cell death ligand 1 (PD-L1) antibody, as monotherapy or in combination with other therapies in the treatment of non-small cell lung cancer (NSCLC)

**DOI:** 10.1186/2051-1426-2-S3-P179

**Published:** 2014-11-06

**Authors:** Julie Brahmer, Ani Balmanoukian, Sarah Goldberg, Sai-Hong Ou, Andrew Blake-Haskins, Joyson Karakunnel, Paul Stockman, Naiyer Rizvi, Scott Antonia

**Affiliations:** 1 Sidney Kimmel Comprehensive Cancer Center at John Hopkins University, Baltimore, MD, USA; 2 The Angeles Clinic and Research Institute, Los Angeles, CA, USA; 3 Yale University, Yale Cancer Center, New Haven, CT, USA; 4 Chao Family Comprehensive Cancer Center, Orange, CA, USA; 5 MedImmune, USA; 6 AstraZeneca Pharmaceuticals, Macclesfield, UK; 7 Memorial-Sloan Kettering Cancer Center, USA; 8 H. Lee Moffit Cancer Center and Research Institute, USA

## Background

MEDI4736 is an engineered human IgG1 that blocks PD-L1 binding to PD-1 and allows T-cells to recognize and kill tumor. MEDI4736 has single-agent activity and potential for further increased activity in combination. A comprehensive development programme is underway in NSCLC, as monotherapy and in combination.

## Methods

NSCLC data from 2 multicentre, open-label Phase I studies are reported. NCT01693562 evaluates safety and efficacy of MEDI4736 administered every 2 or 3 weeks. NCT02000947 evaluates safety and efficacy of MEDI4736 in combination with tremelimumab, a human IgG2 anti-CTLA-4 mAb, at 4-week intervals.

## Results

NCT01693562: As of May 2014, NSCLC cohort included 155 patients (pts) (median age 65 years [33-85], PS 0/1/unknown [25%/73%/2%], median 3 [0-7] prior treatments). Median follow-up: 6 weeks (range 0-67). Treatment-related adverse events (TRAEs): 29% (Grade [Gr] ≥3: 3%); none led to treatment discontinuation. Most frequent TRAEs: fatigue (7%), nausea (5%), and vomiting (5%). No treatment-related colitis any Gr. No treatment-related Gr 3/4 pneumonitis or dyspnea. 58 pts had ≥12 weeks follow-up: 16% had partial response (as early as 6 weeks), duration of response ranged 5-54+ weeks, disease control rate 35%. PD-L1 positivity appears to enrich for response.

**Figure 1:**
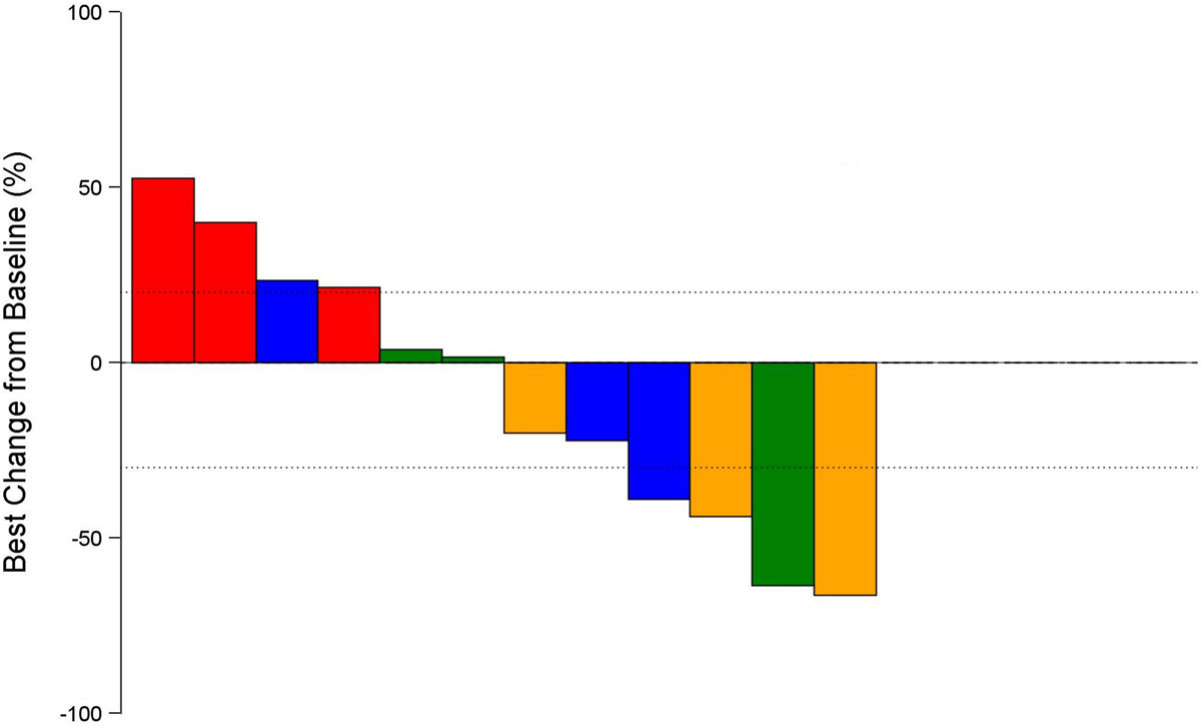
**Red: Cohort1a (MEDI4735/Treme: 3/1); Green: Cohort 2a (MEDI4736/Treme 10/1); Gold: Cohort 3a (MEDI4736/Treme 15/1); Blue: Cohort 3b (MEDI4736/Treme 10/3)**.

NCT02000947: As of April, 2014, 12 pts treated at 4 dose-levels (PS 0-1, median 3 [2-5] prior treatments). No DLTs observed in any cohort during DLT observation period. Most frequent TRAEs: -↑amylase, abdominal pain, arthralgia, colitis, diarrhea, epigastric discomfort, fatigue and nausea. TRAEs ≥Gr 3 noted in 3 pts: Gr 3 - ↑AST/ALT & Gr 5 myasthenia (MG) (n = 1), Gr 3 diarrhea/colitis (n = 1), Gr 4 - ↑amylase (n = 1). TRAEs led to discontinuation in two subjects: Gr 5 MG and Gr3 colitis. In 12 response-evaluable pts (Figure 1), tumor shrinkage at 8 weeks: 0/3 pts cohort 1a; 6/9 pts cohorts 2a and 3a/b; disease control: 7/12 pts. Dose-escalation ongoing; total of 6 dose-levels, including cohort 5a (MEDI4736/Treme: 15/10 mg/kg), has been cleared since cut-off.

## Conclusions

Current safety profile and encouraging early antitumor activity (monotherapy and combination) support continued development in NSCLC. Additional monotherapy NSCLC studies: Phase II 'ATLANTIC' (NCT02087423), Phase III 'PACIFIC' following chemo-radiotherapy (NCT02125461), Combination: Phase I + gefitinib (NCT02088112), and Phase Ib + AZD9291 (NCT02143466). Monotherapy and combination: Phase III 'ARCTIC' vs standard-of-care.

